# Evaluation of a Most Probable Number Method for Detection and Quantification of *Legionella pneumophila*

**DOI:** 10.3390/pathogens11070789

**Published:** 2022-07-12

**Authors:** Chunyan Niu, Yajie Zhang, Yong Zhang

**Affiliations:** Institute for Environmental Health, Beijing Center for Disease Prevention and Control, Beijing 100013, China; niuchunyan86@163.com (C.N.); zy968919@163.com (Y.Z.)

**Keywords:** MPN method, plate culture, *Legionella pneumophila*, cooling water, condensed water, sanitary hot water

## Abstract

The detection and enumeration of *Legionella pneumophila* (*L. pneumophila*) in water is crucial for water quality management, human health and has been a research hotspot worldwide. Due to the time-consuming and complicated operation of the plate culture method, it is necessary to adopt a fast and effective method for application. The present study aimed to comprehensively evaluate the performance and applicability of the MPN method by comparing its qualitative and quantitative results with the GB/T 18204.5-2013 and ISO methods, respectively. The qualitative results showed that 372 samples (53%) were negative for both methods; 315 samples (45%) were positively determined by the MPN method, compared with 211 samples (30%) using GB/T 18204.5-2013. The difference in the detection rate between the two methods was statistically significant. In addition, the quantitative results showed that the concentration of *L. pneumophila* by the MPN method was greater than ISO 11731 and the difference was statistically significant. However, the two methods were different but highly correlated (r = 0.965, *p* < 0.001). The specificity and sensitivity of the MPN method were 89.85% and 95.73%, respectively. Overall, the results demonstrated that the MPN method has higher sensitivity, a simple operation process and good application prospects in the routine monitoring of *L. pneumophila* from water samples.

## 1. Introduction

Legionnaires’ disease (LD) has been listed as one of the emerging infectious diseases in recent decades, seriously threatening people’s lives and health and has become an important public health problem in modern cities around the world. LD is caused by inhaling aerosolized water contaminated with *Legionella*, which is common in both natural freshwater and man-made water supply systems, such as contaminated water distribution systems [1], showers [2,3] spa pools [4], hot springs water [5], faucets [6,7], and cooling towers [8,9,10]. Over the years, outbreaks have continued to occur [11,12,13] and the number of LD cases is increasing each year, according to reports from the US Centers for Disease Control and Prevention and the European Centers for Disease Control and Prevention [14,15]. One species of *Legionella* named *Legionella pneumophila* (*L. pneumophila*) is responsible for more than 90% of all reported cases of legionellosis [16,17]. In conclusion, the contamination of *L. pneumophila* in artificial water in public places around the world is common and severe, which directly leads to the sporadic cases or outbreaks of LD in many countries and regions. Therefore, regular monitoring and detection of *L. pneumophila* from water samples with effective testing methods is essential to maintain public health.

Traditionally, the plate culture method of ISO 11731:2017, utilizing a specific medium (buffer charcoal yeast extract, BCYE), usually with different sets of antibacterial material, has been considered as the gold standard for the detection and enumeration of *Legionella* in water samples. Nevertheless, on account of the slow growth of *Legionella*, it requires 7–10 days for incubation, after which it requires further confirmation with a latex agglutination test specific to *L. pneumophila*. In order to suppress the interference of non-*Legionella* organisms, the plate culture method generally adopts heat treatment or acid treatment which may lead to a lower culturability of *Legionella* in the samples [18]. In addition, some viable but non-culturable bacteria (VBNC) could not be detected by the plate culture method. This is one disadvantage because these VBNC microorganisms may acquire the ability to grow and proliferate under favorable conditions, which may lead to false negative results [19]. In general, the plate culture method has some limitations in daily monitoring. It is time consuming, complex and requires trained staff for sampling and colony recognition. Consequently, there is an urgent need to develop a simpler method to provide reliable qualitative and quantitative results.

Beyond that, some new alternative methods for the quantification of *L. pneumophila* have emerged in recent years. Molecular-based techniques (such as the polymerase chain reaction (PCR)) targeting nucleic acids represent one type of these techniques. The related techniques reported so far include conventional PCR, multiplex PCR, quantitative PCR and real-time PCR [20,21,22,23]. Due to its high reproducibility and quantitative aspects, qPCR has been recognized as a standard method for the detection and quantification of *Legionella* and *L. pneumophila* [24] and was recommended as an alternative method in the 2020 European Drinking Water Directive text [17]. The PCR method has overcome the major drawbacks associated with the use of the plate culture method by detecting *L. pneumophila* (including microorganisms present in a VBNC state) from water samples. There are a number of significant advantages of the PCR method, including high sensitivity, good specificity and short detection time (commonly less than 24 h). Nevertheless, a major drawback is that it does not allow for discrimination between dead and alive cells, as studies have indicated that deoxyribonucleic acid (DNA) may persist after cell death, and this leads to their inability to quantify viable organisms that may pose a risk to human health [25]. Subsequent improvements based on qPCR techniques have been developed, such as Ethidium Monoazide qPCR (EMA-qPCR), Propidium Monoazide qPCR (PMA-qPCR) and droplet digital PCR (ddPCR) which are effectively able to differentiate between viable and dead cells [25,26]. Nevertheless, these PCR-based methods require professional molecular microbiology testing facilities, expensive equipment, complicated processes and trained staff that are not suitable for most water laboratories. Besides this, matrix-assisted laser desorption ionization time of flight mass spectrometry (MALDI-TOF MS) has recently emerged as a rapid species-level microbial identification technique based on ribosomal protein profiling and it was applied to the identification of *L. pneumophila* from water [27,28]. The technology can quickly screen large numbers of colonies and perform a comprehensive analysis of individual colonies in a matter of minutes. Therefore, MALDI-TOF takes less time than PCR. However, colonies that have grown together can no longer be identified directly without subculturing. At the same time, it also requires expensive professional equipment and trained operators. Other methods have also been reported for the detection of *L. pneumophila* in water but are less common, including IMS [29,30], electrochemical genosensors [31] and biosensor-based detection methods [19].

In particular, another *L. pneumophila* testing method is the MPN method which is a type of enzyme substrate technique. The method utilizes a powdered growth medium rich in amino acids, vitamins and other nutrients, which contains a defined growth substrate that selects positively for the growth of *L. pneumophila* only and inhibits the growth of other commensal microorganisms that might be present in the water sample. When the cells turn brown or turbid, it is a sign that *L. pneumophila* is present in water samples [17]. The MPN method could provide a confirmed result in 7 days with rapid sample preparation and analysis. Studies in recent years have indicated that the MPN method is equivalent or better than the plate culture method when used for the determination and evaluation of *L. pneumophila* in potable water and non-potable water [32]. Additionally, many studies have disclosed that the MPN method has a higher sensitivity and specificity compared with the plate culture method [33,34,35,36]. The MPN method has been included in the UK’s Standing Committee of Analysts’ recent update on determining the presence of *Legionella*, which demonstrates that the MPN method is at least as effective as the plate culture method in detecting and enumerating *L. pneumophila* in water samples [37].

To sufficiently evaluate the MPN method, we compared the performance of the MPN method with GB/T 18204.5-2013 (the Chinese regulatory requirements method) [38] and ISO 11731:2017 [39] for the detection and quantification of *L. pneumophila* from three different water samples in this research. The results showed that the MPN method could be used as a valid supplement to the plate culture method to satisfy the testing requirements in China.

## 2. Results

### 2.1. Analysis of Influencing Factors of Sample Preservation in the MPN Method

**Figure 1:**
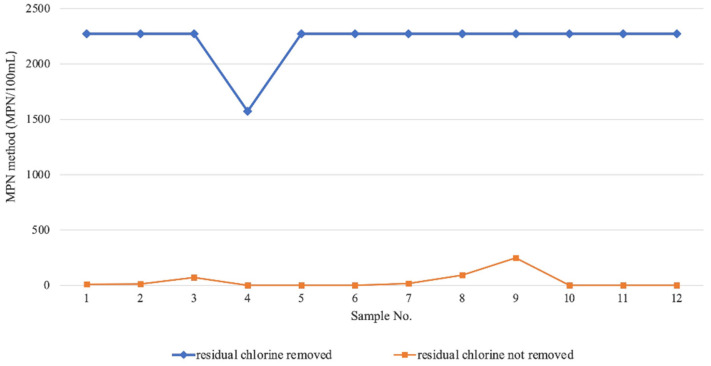
The concentration of *L. pneumophila* was determined by the MPN method under different treatment methods.

In order to further explore the influence of sample storage conditions on the results of the MPN method, a factorial analysis was applied to compare the test results of the same sample under different storage conditions. Specific conditions include sample storage temperature (36 °C, 20 °C and 4 °C), storage time (6 h and 1 h) and different treatment methods (whether to remove residual chlorine in the water). The experimental data were analyzed using Multi-Factor Variance Analysis, and the specific results are shown in Table S1. The results showed that different treatment modes had statistical differences in the impact of *L. pneumophila* test results (“treatment mode”, *p* < 0.001) and the *p* values of interaction between time, treatment method and storage temperature were all >0.05, indicating that there was no interaction between the three factors. Moreover, the concentration of *L. pneumophila* determined by the MPN method under different treatment methods is shown in Figure 1, which supports that removing residual chlorine is more conducive to improving the sensitivity of the detection method.

### 2.2. Comparison with Current Detection Methods

#### 2.2.1. Comparison of Qualitative Test Results with GB/T 182014.5-2013

A comparison of the qualitative detection results of *L. pneumophila* in 696 water samples from five different regions of China was conducted by the MPN method and GB/T 18204.5-2013. In all samples, isolates purified from positive wells obtained by the MPN method were discovered to be predominantly type 1 and type 2, 3, 5, 6, 7 and 8 were also identified. This result is basically consistent with GB/T 182014.5-2013, which indicates that the two methods are similar in the detection of different serogroups.

**Figure 2:**
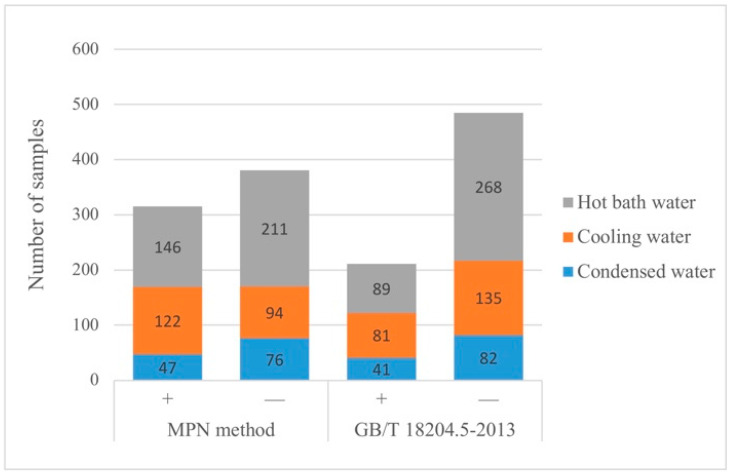
Comparison of the quantitative data from MPN method and ISO method. Note that count for MPN method: log/(MPN/100 mL), ISO method: log/ (CFU/ 100 mL).

Of all the water samples, 372 samples (53%) were negative for both methods, 315 samples (45%) were positive determined by the MPN method, compared with 211 samples (30%) using GB/T 18204.5-2013 (Table 1). McNemar’s test was used to compare the positive rate of *L. pneumophila* between groups, and the statistical results showed *p* < 0.001, indicating that the difference in detection rate between the two methods was statistically significant in this study. This finding agrees with results reported previously for both potable and non-potable water [40]. Moreover, the detection results of *L. pneumophila* in different types of water samples are shown in Table S2 and Figure 2. The McNemar’s test was conducted, and the results showed that there were also statistically significant differences in the detection rates of cooling water (*p* < 0.001), condensed water (*p* < 0.001) and hot bath water (*p* < 0.001) obtained by the two detection methods. Meanwhile, the positive detection rate of the MPN method is about 1.5 times that of GB/T 18204.5-2013 specifically. The above statistical results revealed that the MPN method was more sensitive than GB/T 18204.5-2013 in this study for water samples.

It may be because the LOD of the MPN method was lower than GB/T 18204.5-2013 that more positive samples could be detected, leading to the inconsistent distribution of positive samples in the two methods. Specifically, there were 113 samples that were negative for GB/T 18204.5-2013 but were positive for the MPN method, while only 9 samples were positive for GB/T 18204.5-2013 and negative for the MPN method. The main difference lies in cooling water samples and hot bath water samples (Table S2). In this study, the 113 water samples were further analyzed for confirmatory test (methods described in Section 4.3), and the results demonstrated that 71 of them were positive for *L. pneumophila*, which indicates that the GB/T 18204.5-2013 has a relatively high false negative rate (71/113) and further certified that the sensitivity of the MPN method for the determination of *L. pneumophila* in water was higher than that of GB/T 18204.5-2013.

We evaluated the sensitivity and specificity of the MPN method with GB/T 18204.5-2013 as a reference method which is currently considered as the gold standard for detecting *L. pneumophila* in water in China. After eliminating the false negative results of GB/T 18204.5-2013, the specificity of the MPN method was 89.85% (372/(485 − 71)), while its sensitivity was 96.81% ((202 + 71)/(211 + 71)).

#### 2.2.2. Comparison of Quantitative Test Results with ISO 11731:2017

The MPN method and ISO method were used for quantitative detection in 170 artificially contaminated water samples from five different regions in China. After the logarithmic processing of quantitative data, the Wilcoxon rank-sum test was used for statistical analysis, and the results showed that the difference between the two methods was statistically significant (*p* < 0.001). In addition, the median of MPN method (3.89 log MPN/100 mL) was larger than that of ISO method (3.33 log CFU/100 mL) (Table S3), with concentrations of up to 4.92 log MPN/100 mL for the MPN method and up to 4.44 log CFU/100 mL for the ISO method, respectively.

**Figure 3:**
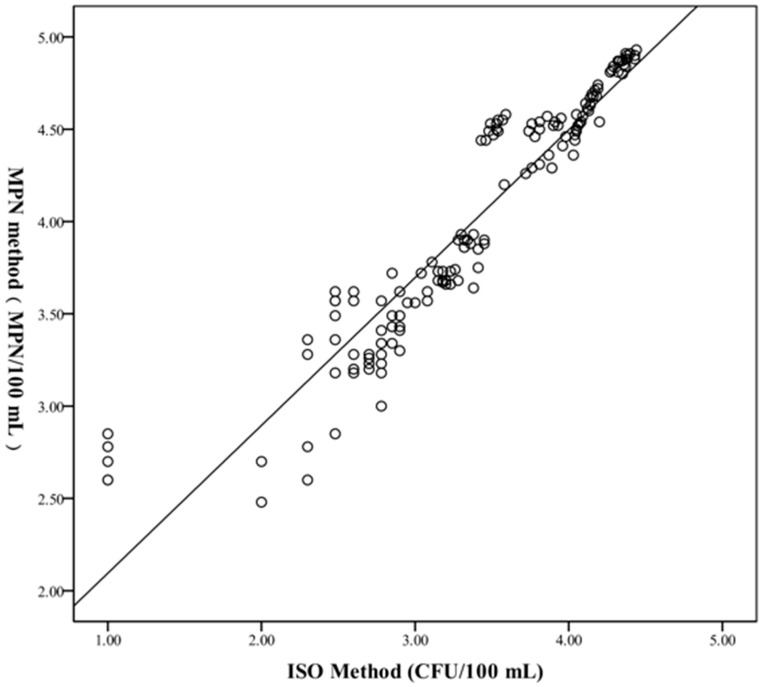
Comparison of the quantitative data from MPN method and ISO method. Count in y-axis: log/(MPN/100 mL) for MPN method or log/(CFU/100 mL) for ISO method.

Meanwhile, Spearman’s correlation analysis was conducted on the above quantitative data to analyze the correlation between the two methods and the results indicated that although the results of the two methods were different, they were highly correlated (r = 0.965, *p* < 0.001). The scatter plot of the MPN method with the ISO method is shown in Figure 3.

**Figure 4:**
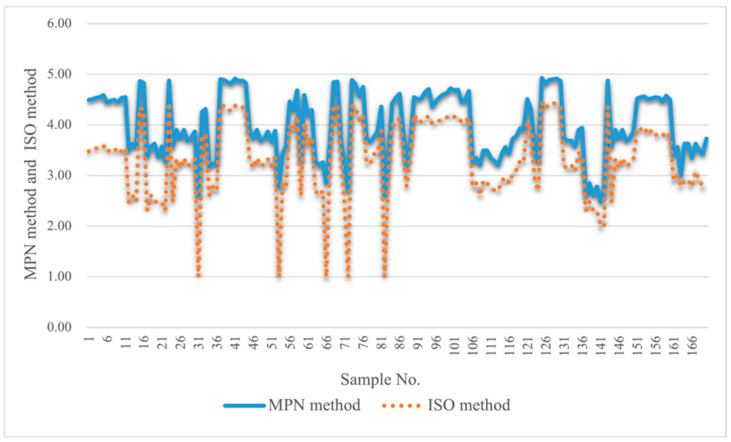
Detection data of *L. pneumophila* in different water samples by MPN method and GB/T 18204.5-2013.

Multiple analysis results showed that the MPN method had a higher sensitivity than the ISO method in detecting *L. pneumophila* in these water samples. Firstly, the linear regression line was log MPN = 1.1685 × log ISO which revealed that the MPN method’s detection was slightly higher. Second, the line graph (Figure 4) of the quantitative results of the two methods showed that the bacterial counts of the same sample by the MPN method were all higher than those by ISO method in 170 water samples. Finally, in the experimental process, it was discovered that when the labeled sample was diluted to 10^−5^–10^−6^ CFU/100 mL, *L. pneumophila* could be detected by the MPN method, while it could not be detected by the ISO method. Hence, this proved that the MPN method was more sensitive than the ISO method in the determination of *L. pneumophila* in water.

## 3. Discussion

Considering that the Chinese Hygienic Indicators and Limit Requirements for Public Places (GB 37488-2019) only set limit requirements for *L. pneumophila* in cooling water, condensed water of centralized air conditioning and ventilation systems and sanitary hot water, three types of water samples were selected for inspection in this research. In order to better study the applicability of the MPN method, the range of water sample types can be further expanded in future experiments.

In Legiolert^TM^’s operating instructions, there are two slightly different treatments for potable and non-potable water. In studies in the literature [41,42], measures of cooling water and condensation water were carried out in accordance with the method of non-potable water pretreatment, which was consistent with our results. In the preliminary experiment, we compared the two pretreatment methods of these three kinds of water samples, observed the difference of their detection results, and finally chose the non-potable water pretreatment program. Nonetheless, for sanitary hot water, the method in the literature of a selected potable water pretreatment program [43] was not the same in our research, which may be caused by the different water quality in different countries. This indicates that when choosing the MPN method, we should select the appropriate pretreatment method according to the specific situation of regional water quality.

In this study, it was discovered that the *L. pneumophila* content detected by the plate culture method is often lower than the MPN method, which may be due to the following reasons: 1. It was discovered that the MPN method had strong anti-interference ability against other microorganisms other than *L. pneumophila*. 2. *L. pneumophila* may be more suitable for growing and reproducing on the liquid medium of the MPN method than the solid medium of the plate culture method. 3. In this method, there were no centrifugation or filtration steps to destroy bacteria. In contrast, the plate culture method requires a variety of pretreatment methods to minimize the interference of miscellaneous bacteria, such as heat treatment, acid treatment and filtration, which improve the detection rate of *L. pneumophila* to a certain extent. Nevertheless, it is difficult to acquire complete selectivity for *L. pneumophila* without decreasing its recovery rate while suppressing the growth of non-pneumophila species. To sum up, the MPN method yielded significantly higher counts of *L. pneumophila* from water samples than ISO 11731 and retrieved a greater number of positive samples than ISO 11731 in the same samples. Consequently, the MPN method has a tendency to detect more *L. pneumophila* than the standard method in water samples.

In any case, in all kinds of water samples, it is difficult to avoid the existence of a variety of miscellaneous bacteria that may interfere with the detection of *L. pneumophila*. Even the use of the MPN method cannot entirely avoid this. This may be why the MPN method produces a small number of false positive samples, which has also been observed in other studies [34,42]. In this study, it was found that the elimination of residual chlorine could effectively improve the detection ability of *L. pneumophila* by the MPN method, which was consistent with the opinion of a reported article [35], because chlorine residues decrease the culturability of *L. pneumophila*.

In addition, quality control procedures also need to be mentioned. Due to the simple operation procedure of the MPN method, there are fewer steps to be performed in quality control. Only the MPN method reagent and the pretreatments for non-potable samples were tested.

## 4. Materials and Methods

### 4.1. Samples

A total of 696 water samples were used, including cooling water (216), condensed water (123) and sanitary hot water (357). They originated from cooling towers, evaporative condensers, showers and hot tap water. In view of the low content of *L. pneumophila* in the investigated samples, we selected 170 water samples that were not detected by *L. pneumophila* for artificial contamination at three different concentration levels, in order to better evaluate the quantitative performance of the MPN method. Specifically, it contains cooling water (70), condensed water (50) and hot bath water (50).

According to the protocol contained in the Chinese guidelines [44], *L. pneumophila* water samples should be kept at room temperature under dark, and sent to the laboratory to be analyzed within 48 h.

### 4.2. MPN Method

The MPN method is based on the detection of *L. pneumophila* cells reacting with the enzyme substrate to make the solution brown or turbid. The Legiolert^TM^ (IDEXX Laboratories, Inc., Westbrook, ME, USA) protocol for non-potable water detection was adopted in this paper. Briefly, a pretreatment solution which could inhibit the growth of miscellaneous bacteria was first prepared by adding 100 mL sterile deionized water to a container with a powdered pretreatment reagent and shaking was performed until it was completely dissolved. Then, a 2 mL water sample was added to 2 mL of the pretreatment solution, mixed, and incubated at room temperature for 1 min. After incubation, 2 mL of the solution was promptly transferred to the reagent, which was a nutrient solution containing enzyme substrates, made from Legiolert^TM^ blister pack dissolved in 100 mL sterile water and fully mixed. The solution was poured into the 96-well Quanti-Tray^®^ and sealed by a Sealer Plus (IDEXX Laboratories, Inc., Westbrook, MA, USA) immediately. The Quanti-Trays^®^ were incubated at 37 ± 0.5 °C for 7 days with humidity. If the wells of Quanti-Tray^®^ were more brown or turbid than the negative control, they were considered to be positive. The number of all positive wells was recorded, and the result was determined according to the MPN table. Then, the counting unit was expressed in MPN/100 mL.

### 4.3. Confirmatory Test

According to the manufacturer’s instructions, the MPN method is a specific test for *L. pneumophila*. However, in order to test the specificity of this method, further confirmatory tests were carried out on positive wells according to the following principles. The Quanti-Tray^®^ has 6 big wells and 90 small ones. If the number of positive wells was less than 10, all of them were confirmed; if the number of positive wells was greater than 10, 10 wells (including all big wells and randomly selected small ones) were chosen first, and 25% of the remaining positive wells were randomly identified. Confirmation was performed by extracting a 10 μL suspension with a sterile syringe on both BCYE and BCYE-cys. After incubation at 36 ± 1 °C for 48–72 h, the colonies were regarded as *L. pneumophila* if they grew on BCYE but failed to grow on BCYE-cys. Subsequently, confirmed isolates were tested by latex agglutination to determine the species and serotype of the isolates.

### 4.4. ISO 11731:2017

The plate culture method is represented by ISO 11731. Briefly, 500 mL of water samples were filtered through a 0.45-μm pore-sized membrane and the membrane was transferred to the original water sample and dissolved by vortexing. Subsequently, one concentrated water sample, one acid-treated concentrated water sample, and one heat-treated concentrated water sample were inoculated on three glycine, vancomycin, polymyxin B, and cycloheximide (GVPC) agar plates, respectively. The amount for inoculation was 100 μL. All the plates were incubated at 36 ± 1 °C for 7–10 d. Three colonies of each putative *Legionella* colonies were selected for subculture to further confirm the type and serotype of the isolates. The confirmatory method was described above (Section 4.3). A culture plate with the assumed maximum number of *Legionella* colonies was selected from three sample culture plates with different pretreatment methods to the final result, and the counting unit was expressed in CFU/100 mL.

### 4.5. GB/T 18204.5-2013

This method is a qualitative method for the detection of *L. pneumophila* in environmental water samples in China, and it basically conforms to ISO 11731:2017 in terms of sample pretreatment and culture conditions. The biggest difference between the two methods is that GB/T 18204.5-2013 only needs to confirm the suspected *L. pneumophila* colonies in the sample but does not need to count.

### 4.6. Data Analysis

All data processing was performed by the WPS office or SPSS 19.0 (SPSS Inc., Chicago, IL, USA). Before statistical analysis, the data were log transformed. In view of previous documentation [36], MPN units obtained with the MPN method were equivalent to CFU units obtained with ISO 11731.

In this study, we adopted the factorial analysis method to compare the test results of the same sample under different storage conditions. All qualitative data were analyzed by a McNemar’s binomial test to evaluate the differences between the frequencies of positive and negative samples by the test methods. All quantitative data were found to be of a non-normal distribution using the Shapiro–Wilk test (*p* < 0.0001); therefore, the sensitivity difference between the two methods was also determined by non-parametric Wilcoxon signed-rank test. In addition, Spearman’s correlation analysis was also conducted on the above quantitative results to test the correlation between the two methods.

## 5. Conclusions

The present study indicated the MPN method provides some advantages over the plate culture method used to detect and enumerate *L. pneumophila* in water samples. These advantages include good sensitivity and selectivity, user acceptability, a simplified protocol that requires less time and fewer resources, the ability to employ smaller sample volumes to obtain the same quantification and a clear positive signal which was easy to read and needed little to no interpretation. However, the MPN method also has its limitations in the detection of *L. pneumophila* from water. For example, it has the potential to produce false positives as demonstrated in this study. After all, this is a technology based on bacterial culture, so it takes time to detect it, and seven days is not short compared to molecular technology. To sum up, the MPN method represents a significant improvement in the qualitative and quantitative detection of *L. pneumophila* from water which could increase the positive rate of culture and the possibility of isolating *L. pneumophila*. Consequently, it can be used as an initial screening method for unknown water samples. It is also a simple and effective routine monitoring method for *L. pneumophila*.

## Figures and Tables

**Table 1:** McNemar’s test analysis of the results for detection of *L. pneumophila* for MPN method and GB/T 18204.5-2013.

MPN Method	GB/T 18204.5-2013		Total	χ^2^-Value	*p*-Value
	+	−			
+	202	113	315	311.388	<0.001
−	9	372	381		
Total	211	485	696		

## Data Availability

Not applicable.

## Supplementary Materials

The following supporting information can be downloaded at: https://www.mdpi.com/article/10.3390/pathogens11070789/s1, Table S1: Effects of different storage conditions on test results; Table S2: Detection results of *L. pneumophila* in different water samples by MPN method and GB/T 18204.5-2013; Table S3: Outcome of statistical analysis of the paired *L. pneumophila* counts from water samples by MPN method and ISO method.

## References

[1] Hines S.A., Chappie D.J., Lordo R.A., Miller B.D., Janke R.J., Lindquist H.A., Fox K.R., Ernst H.S., Taft S.C. (2014). Assessment of relative potential for Legionella species or surrogates inhalation exposure from common water uses. Water Res..

[2] Hayes-Phillips D., Bentham R., Ross K., Whiley H. (2019). Factors influencing Legionella contamination of domestic household showers. Pathogens.

[3] Zeng L.Z., Liao H.Y., Luo L.Z., Sen H.S., Qin T., Zhou H.J., Li H.X., Da Li C.H., Chen J.P. (2019). An investigation on the molecular characteristics and intracellular growth ability among environmental and clinical isolates of legionella pneumophila in sichuan province, China. Biomed. Environ. Sci..

[4] Leoni E., Catalani F., Marini S., Dallolio L. (2018). Legionellosis Associated with Recreational Waters: A Systematic Review of Cases and Outbreaks in Swimming Pools, Spa Pools, and Similar Environments. Int. J. Environ. Res. Public Health.

[5] Zhang L.Y., Li Y., Wang X., Shangguan Z.H., Zhou H.J., Wu Y., Wang L., Ren H., Hu Y., Lin M. (2017). High Prevalence and Genetic Polymorphisms of Legionella in Natural and Man-Made Aquatic Environments in Wenzhou, China. Int. J. Environ. Res. Public Health.

[6] Gavalda L., Garcia-Nunez M., Quero S., Gutierrez-Milla C., Sabria M. (2019). Role of hot water temperature and water system use on Legionella control in a tertiary hospital: An 8-year longitudinal study. Water Res..

[7] Xiao G.W., Chen G.M., Xiao Y.X., Chen S.J., Xie W.Q., Ding J.M. (2020). Contamination status of Legionella in partial public places of Meizhou city. J. Trop. Med..

[8] Jiang L.X., Zhao S.H., Cai X., Mu D.G., Zhang X., Kang J., Zhao L., Chen Y. (2020). Sequence-based typing of clinical and environmental Legionella pneumophila isolates in Shenyang, China. Enferm. Infecc. Microbiol. Clin..

[9] Thornley C.N., Harte D.J., Weir R.P., Allen L.J., Knightbridge K.J., Wood P.R.T. (2017). Legionella longbeachae detected in an industrial cooling tower linked to a legionellosis outbreak, New Zealand, 2015; possible waterborne transmission?. Epidemiol. Infect..

[10] Wang L., Duan G. (2017). Legionella Pneumophila Contamination in Water System in Public Places of Chongqing. J. Prev. Med. Inf..

[11] Crook B., Willerton L., Smith D., Wilson L., Poran V., Helps J., McDermott P. (2020). Legionella risk in evaporative cooling systems and underlying causes of associated breaches in health and safety compliance. Int. J. Hyg. Environ. Health.

[12] Nakamura I., Amemura-Maekawa J., Kura F., Kobayashi T., Sato A., Watanabe H., Matsumoto T. (2020). Persistent Legionella contamination of water faucets in a tertiary hospital in Japan. Int. J. Infect. Dis..

[13] Puri S., Boudreaux-Kelly M., Walker J.D., Clancy C.J., Decker B.K. (2020). Clinical Presentation of Community-Acquired Legionella Pneumonia Identified by Universal Testing in an Endemic Area. Int. J. Environ. Res. Public Health.

[14] Beauté J., The European Legionnaires’ Disease Surveillance Network (2017). Legionnaires’ disease in Europe, 2011 to 2015. Euro Surveill..

[15] Garrison L.E., Kunz J.M., Cooley L.A., Moore M.R., Lucas C., Schrag S., Sarisky J., Whitney C.G. (2016). Vital signs: Deficiencies in environmental control identified in outbreaks of legionnaires’ disease: North America, 2000–2014. Morb. Mortal. Wkly. Rep..

[16] European Centre for Disease Prevention and Control (2016). Legionnaires’ Disease in Europe, 2014.

[17] Walker J.T., McDermott P. (2021). Confirming the Presence of Legionella pneumophila in Your Water System: A Review of Current Legionella Testing Methods. J. AOAC Int..

[18] Leoni E., Legnani P.P. (2001). Comparison of selective procedures for isolation and enumeration of Legionella species from hot water systems. J. Appl. Microbiol..

[19] Islam M.A., Hassen W.M., Tayabali A.F., Dubowski J.J. (2021). Short Ligand, Cysteine-Modified Warnericin RK Antimicrobial Peptides Favor Highly Sensitive Detection of Legionella pneumophila. ACS Omega.

[20] Ahmed S., Liwak-Muir U., Walker D., Zoldowski A., Mears A., Golovan S., Mohr S., Lem P., Harder C. (2019). Validation and in-field testing of a new on-site qPCR system for quantification of Legionella pneumophila according to ISO/TS 12869:2012 in HVAC cooling towers. J. Water Health.

[21] Boss R., Baumgartner A., Kroos S., Blattner M., Fretz R., Moor D. (2018). Rapid detection of viable legionella pneumophila in tap water by a qPCR and RT-PCR-based method. J. Appl. Microbiol..

[22] Eble D., Gehrig V., Schubert-Ullrich P., Kppel R., Füchslin H.P. (2021). Comparison of the culture method with multiplex PCR for the confirmation of Legionella spp. and Legionella pneumophila. J. Appl. Microbiol..

[23] Sawczyn-Domańska A. (2021). Detection of Legionella spp. and occurrence of virulence genes: Lvh, rtxA and enhC in water samples from artificial water systems. Ann. Agric. Environ. Med..

[24] (2012). ISO/TS 12869. Water Quality—Detection and Quantification of *Legionella* spp. and/or *Legionella* pneumophila by Concentration and Genic Amplification by Quantitative Polymerase Chain Reaction (qPCR). https://www.iso.org/standard/52079.html.

[25] Reyneke B., Ndlovu T., Khan S., Khan W. (2017). Comparison of EMA-, PMA- and DNase qPCR for the determination of microbial cell viability. Appl. Microbiol. Biotechnol..

[26] Falzone L., Gattuso G., Lombardo C., Lupo G., Salmeri M. (2020). Droplet digital PCR for the detection and monitoring of legionella pneumophila. Int. J. Mol. Med..

[27] Pascale M.R., Mazzotta M., Salaris S., Girolamini L., Grottola A., Simone M.L., Cordovana M., Bisognin F., Dal Monte P., Bucci Sabattini M.A. (2020). Evaluation of MALDI-TOF Mass spectrometry in diagnostic and environmental surveillance of legionella species: A comparison with Culture and Mip-Gene Sequencing Technique. Front. Microbiol..

[28] Trnková K., Kotrbancová M., Špaleková M., Fulová M., Boledovičová J., Vesteg M. (2018). MALDI-TOF MS analysis as a useful tool for an identification of Legionella pneumophila, a facultatively pathogenic bacterium interacting with free-living amoebae: A case study from water supply system of hospitals in Bratislava (Slovakia). Exp. Parasitol..

[29] Díaz-Flores Á., Montero J.C., Castro F.J., Alejandres E.M., Bayón C., Solís I., Fernández-Lafuente R., Rodríguez G. (2015). Comparing methods of determining Legionella spp. in complex water matrices. BMC Microbiol..

[30] Keserue H.A., Cornillie N., Ehlert A.K., Mills D.C., Morger D., Piffaretti A., Schaffhauser D.F., Schwyzer I.I. (2021). Validation of the Legionella pneumophila SG1 DETECT Kit for Quantification of Legionella pneumophila Serogroup 1 Bacteria in Potable Waters, Process Waters, and Surface Waters: AOAC Performance Tested Methodsm 052002. J. AOAC Int..

[31] Olabarria G., Eletxigerra U., Rodriguez I., Bilbao A., Berganza J., Merino S. (2020). Highly sensitive and fast *Legionella* spp. in situ detection based on a loop mediated isothermal amplification technique combined to an electrochemical transduction system. Talanta.

[32] Scaturro M., Buffoni M., Girolamo A., Cristino S., Ricci M.L. (2020). Performance of Legiolert test vs. ISO 11731 to Confirm *Legionella pneumophila* Contamination in Potable Water Samples. Pathogens.

[33] Checa J., Carbonell I., Manero N., Martí I. (2021). Comparative study of Legiolert with ISO 11731-1998 standard method-conclusions from a Public Health Laboratory. J. Microbiol. Methods.

[34] Isabelle B. (2019). Comparison of Legiolert and a Conventional Culture Method for Detection of Legionella pneumophila from Cooling Towers in Québec. J. AOAC Int..

[35] Boczek L.A., Tang M., Formal C., Lytle D., Ryu H. (2021). Comparison of two culture methods for the enumeration of Legionella pneumophila from potable water samples. J. Water Health.

[36] Sartory D.P., Spies K., Lange B., Schneider S., Langer B. (2017). Evaluation of a most probable number method for the enumeration of legionella pneumophila from potable and related water samples. Lett. Appl. Microbiol..

[37] SCA (2020). The Determination of Legionella Bacteria in Waters and Other Environmental Samples (2020)–Part 2–Culture Methods for Their Detection and Enumeration. https://www.researchgate.net/publication/346446532_The_determination_of_Legionella_bacteria_in_waters_and_other_environmental_samples_2020_-Part_2_-Culture_Methods_for_their_detection_and_enumeration_Methods_for_the_Examination_of_Waters_and_Associate.

[38] State General Administration of the People’s Republic of China for Quality Supervision and Inspection and Quarantine (AQSIQ) (2013). Examination Methods for Public Places―Part 5: Central Air Conditioning Ventilation System GB/T 18204.5-2013. AQSIQ, China. http://www.gb688.cn/bzgk/gb/newGbInfo?hcno=45F2B6F3F56D68AE22080B7E61F91411.

[39] (2017). Water Quality—Enumeration of Legionella.

[40] Monteiro S.N., Robalo A.M., Santos R.J. (2021). Evaluation of Legiolert for the Detection of Legionella pneumophila and Comparison with Spread-Plate Culture and qPCR Methods. Curr. Microbiol..

[41] Petrisek R., Hall J. (2018). Evaluation of a most probable number method for the enumeration of legionella pneumophila from north american potable and nonpotable water samples. J. Water Health.

[42] Rech M.M., Swalla B.M., Dobranic J.K. (2018). Evaluation of Legiolert for Quantification of Legionella pneumophila from Non-potable Water. Curr. Microbiol..

[43] Inoue H., Baba M., Tayama S. (2020). Evaluation of Legiolert for Quantification of Legionella pneumophila from Bath Water Samples. Biocontrol Sci..

[44] State General Administration of the People’s Republic of China for Quality Supervision and Inspection and Quarantine (AQSIQ) (2013). Examination Methods for Public Places―Part 6: Technical Specifications of Health Monitoring GB/T 18204.6-2013, AQSIQ, China. http://www.gb688.cn/bzgk/gb/newGbInfo?hcno=E92DD7EEC2A692420E31074E7A09D194.

