# Supplementary material for: Evaluation of a Most Probable Number Method for Detection and Quantification of Legionella pneumophila
Source: Pathogens. 2022 Jul 12;11(7):789. doi: 10.3390/pathogens11070789 (PMC9324539; doi:10.3390/pathogens11070789)
Supplement: Supplementary file 1 [file pathogens-11-00789-s001.zip › Table S1.pdf]

Table S1 Effects of different storage conditions on test results

Source	Type III Sum of Squares	df	Mean Square	F	Sig.
Corrected Model	29.710a	11	2.701	10.692	0.000
Intercept	153.217	1	153.217	606.553	0.000
Time	0.002	1	0.002	0.007	0.933
Treatment methods	28.306	1	28.306	112.055	0.000
Temperature	0.111	2	0.056	0.22	0.804
Time * Treatment methods	0.115	1	0.115	0.457	0.506
Time * Temperature	0.462	2	0.231	0.915	0.415
Treatment methods * Temperature	0.203	2	0.101	0.401	0.674
Time * Treatment methods * Temperature	0.488	2	0.244	0.966	0.395
Error	5.81	23	0.253		
Total	195.378	35			
Corrected Total	35.52	34			
