# Supplementary material for: Evaluation of a Most Probable Number Method for Detection and Quantification of Legionella pneumophila
Source: Pathogens. 2022 Jul 12;11(7):789. doi: 10.3390/pathogens11070789 (PMC9324539; doi:10.3390/pathogens11070789)
Supplement: Supplementary file 1 [file pathogens-11-00789-s001.zip › Table S2.pdf]

Table S2 Detection results of *L. pneumophila* in different water samples by MPN method and GB/T 18204.5-2013

Water samples	MPN method		Detection Rate	GB/T 18204.5-2013		Detection Rate	χ^2 -Value	*P* -*V*laue
	+	-	（%）	+	-	（%）		
Condensed water	47	76	38.21	41	82	33.33	93.81	0.000
Cooling water	122	94	56.48	81	135	37.50	74.90	0.000
Hot bath water	146	211	40.90	89	268	24.93	66.80	0.000
