# Supplementary material for: Evaluation of a Most Probable Number Method for Detection and Quantification of Legionella pneumophila
Source: Pathogens. 2022 Jul 12;11(7):789. doi: 10.3390/pathogens11070789 (PMC9324539; doi:10.3390/pathogens11070789)
Supplement: Supplementary file 1 [file pathogens-11-00789-s001.zip › Table S3 .pdf]

Table S3 Outcome of statistical analysis of the paired *L. pneumophila* counts from water samples by MPN method and ISO method

Methods	N	Percentiles			*P* -Value
		25th	50th (Median)	75th	
MPN	170	3.49	3.89	4.54	0.000
ISO	170	2.85	3.33	4.04	
